# Population-based virucidal phthalocyanine gargling/rinsing protocol to reduce the risk of coronavirus disease-2019: a community trial

**DOI:** 10.3205/dgkh000426

**Published:** 2022-12-06

**Authors:** Verônica Caroline Brito-Reia, Roosevelt da Silva Bastos, Fabiano Vieira Vilhena, Heitor Marques Honório, Lucas Marques da Costa Alves, Paulo Frazão, Paulo Sérgio da Silva Santos

**Affiliations:** 1 Department of Surgery, Stomatology, Pathology, and Radiology, Bauru School of Dentistry, University of São Paulo, Bauru, Brazil; 2 Department of Pediatric Dentistry, Orthodontics and Public Health, Bauru School of Dentistry, University of São Paulo, Bauru, Brazil; 3 Oral Health & Technologies, Bauru, Brazil; 4 Hospital Estadual de Bauru, Bauru, Brazil; 5 Department Public Health School at University of São Paulo, São Paulo, Brazil

**Keywords:** COVID-19, severe acute respiratory syndrome coronavirus 2, antiviral mouthwash, population-based study, phthalocyanine derivate

## Abstract

**Aim:**

In this community trial, the objective was to evaluate the incidence of coronavirus disease-2019 (COVID-19) cases in two similar communities in three distinct phases: 1 (before the intervention), 2 (during the intervention), and 3 (after the intervention).

**Methods:**

The test community received the oral antiseptic intervention (experimental), while the control community did not. The official information agency (“Statewise System for Data Analysis”) provided the number of confirmed COVID-19 cases. Data were analyzed according to the three phases per epidemiological week (epi) using the R Core Team (2021) program. The relative risk and 95% confidence intervals between the cumulative incidence values of the test and control communities were calculated for each period. In the test community, a total of 995 residents over 10 years of age received two bottles containing 600 ml of mouthwash containing antiviral phthalocyanine derivative (APD). The participants were asked to gargle/rinse with of 5 mL of the mouthwash containing ADP 3 to 5 times a day, for 1 min, until the bottles were empty.

**Results:**

In phases 1 and 3, the disease risk between the two communities did not differ significantly (p>0.05), while in phase 2, the disease risk was 54% lower in the test community than in the control community.

**Conclusion:**

The use of the APD mouthwash protocol seems to reduce the COVID-19 incidence at the population level, and further studies are needed to confirm its protective effect under more precisely controlled conditions.

## Zusammenfassung

**Zielsetzung:**

In einer Bevölkerungsstudie sollte das Auftreten von COVID-19 in zwei vergleichbaren Gemeinden in drei verschiedenen Phasen untersucht werden: Phase 1 vor, Phase 2 während und Phase 3 nach der Intervention.

**Methode:**

Die Testpopulation erhielt die orale antiseptische Intervention, die Kontrollpopulation dagegen nicht. Die amtliche Informationsstelle lieferte die Zahl der bestätigten COVID-19 Fälle. Die Daten wurden entsprechend den drei Phasen pro epidemiologischer Woche (epi) mit dem Programm R Core Team (2021) analysiert. Das relative Risiko und die 95%-Konfidenzintervalle zwischen den kumulativen Inzidenzwerten der Test- und Kontrollpopulation wurden für jeden Zeitraum berechnet. In der Testpopulation erhielten 995 Einwohner im Alter >10 Jahre zwei Flaschen mit je 600 ml Mundwasser mit Gehalt an dem antiviralen Phthalocyanin-Derivat (APD). Die Teilnehmer wurden gebeten, 3 bis 5 Mal täglich 1 min lang mit 5 ml des Mundwassers zu gurgeln/zu spülen, bis die Flaschen aufgebraucht sind.

**Ergebnisse:**

In den Phasen 1 und 3 unterschied sich das Erkrankungsrisiko zwischen den beiden Bevölkerungsstichproben nicht signifikant (p>0,05), während in Phase 2 das Erkrankungsrisiko in der Testpopulation 54% niedriger war als in der Kontrollpopulation.

**Schlussfolgerung:**

Das viruzide APD-Mundspülprotokoll scheint die COVID-19-Inzidenz auf Bevölkerungsebene zu verringern, jedoch sind weitere Studien erforderlich, um die Schutzwirkung unter genauer kontrollierten Bedingungen zu bestätigen.

## Introduction

At the end of 2019, severe acute respiratory syndrome coronavirus 2 (COVID-19) emerged in Wuhan, China, and rapidly spread to other countries, starting a pandemic with high hospitalization and lethality (COVID-19) [1], [2], [3], [4]. Faced with this situation, world leaders were expected to take primary prevention measures (health promotion and specific protection) to prevent the collapse of health systems. For this reason, the World Health Organization developed a strategic plan to block COVID-19 as follows:

1. mobilize all sectors and communities,
2. control sporadic cases and clusters and prevent community transmission,
3. suppress community transmission,
4. reduce mortality by providing appropriate clinical care for those affected by COVID-19, and
5. develop safe and effective vaccines and therapeutics [4].

The protective effect of vaccines against SARS-CoV-2 has been assessed on the population in order to prevent infections and symptoms, despite variants of the coronavirus [4]. However, wearing masks, hand hygiene, and social distancing in conjunction with the use of mouthwashes has been recommended as an important preventive measure against contamination with and dissemination of SARS-CoV-2 [5], [6], [7], [8], [9]. The recommended use of antiviral oral and nasal care products is based on the knowledge that respiratory infections, such as COVID-19, favor the adhesion and replication of pathogens to the nasal and oral mucosa and, consequently, to the respiratory tract [5]. In this sense, the use of gargling/rinsing protocols with oral antiseptic solutions has been indicated both as an adjuvant therapy in the treatment of viral infections and as a prophylactic strategy to minimize the spread of the disease [2], [5], [8], [10], [11], [12].

Phthalocyanine is an antimicrobial compound that selectively destroys viruses, bacteria, and other pathogens, with oxidizing potential and direct binding to SARS-CoV-2 ribonucleic acid (RNA) [13]. Its derivative is composed of oxidants, which promote self-activation and continuous production of reactive oxygen species in the presence of molecular oxygen [14]. The molecules of this compound thus cause oxidative stress to microorganisms, leading to their inactivation and blocking the growth of infectious particles through contact with oral antiseptics at the time of rinsing/gargling. A mouthwash containing an anti-SARS-CoV-2 phthalocyanine derivative has been indicated during the pandemic, since COVID-19-positive patients showed clinical improvement and less severe illness after using a gargle/rinse protocol [6], [7], [8], [9]. 

Although the literature has reported the action of some antiviral mouthwashes to help against SARS-CoV-2 [12] in clinical trails [15], [16], [17], [18], [19], no study has explored its effect on the incidence of COVID-19 in populations. Here, we evaluated the ability of an antiviral phthalocyanine-derivative (APD) mouthwash to reduce the risk of COVID-19 in a Brazilian community.

## Materials and methods

This controlled before-and-after community trial evaluated the cumulative incidence of COVID-19 cases from 04/11/2020 to 05/15/2021 in two similar municipalities, Uru and Itaju, located in a Brazilian state with the highest number of accumulated cases. Both municipalities belong to the same mesoregion within São Paulo state, Brazil, and have similar socio-demographic profiles according to official data from the Brazilian Institute of Geography and Statistics [20]. The state of São Paulo is divided into 17 mesoregions, and COVID-19 arose at different times and behaved in different ways in each of them. The authors followed the epidemic course in the mesoregion of Bauru, and the urban population living in Uru was invited to participate in the study in accordance with the principles of the Declaration of Helsinki, ethical standards of human experimentation, and biosafety protocols with the approval of the Human Research Ethics Committee (CAAE 39327120.3.0000.5417). It was also registered at the Brazilian Clinical Trial Register (RBR-6c9xnw3). Uru was selected to receive the APD mouthwash protocol, since this community had a major incidence of COVID-19 (>50 cases/1,000 inhabitants) at the beginning of the study and because the health authorities offered prompt support. To compare the estimates, we selected as the control the population of Itaju, which had a similar epidemic situation.

### Setting and epidemiological characteristics of municipalities

**Figure 1:**
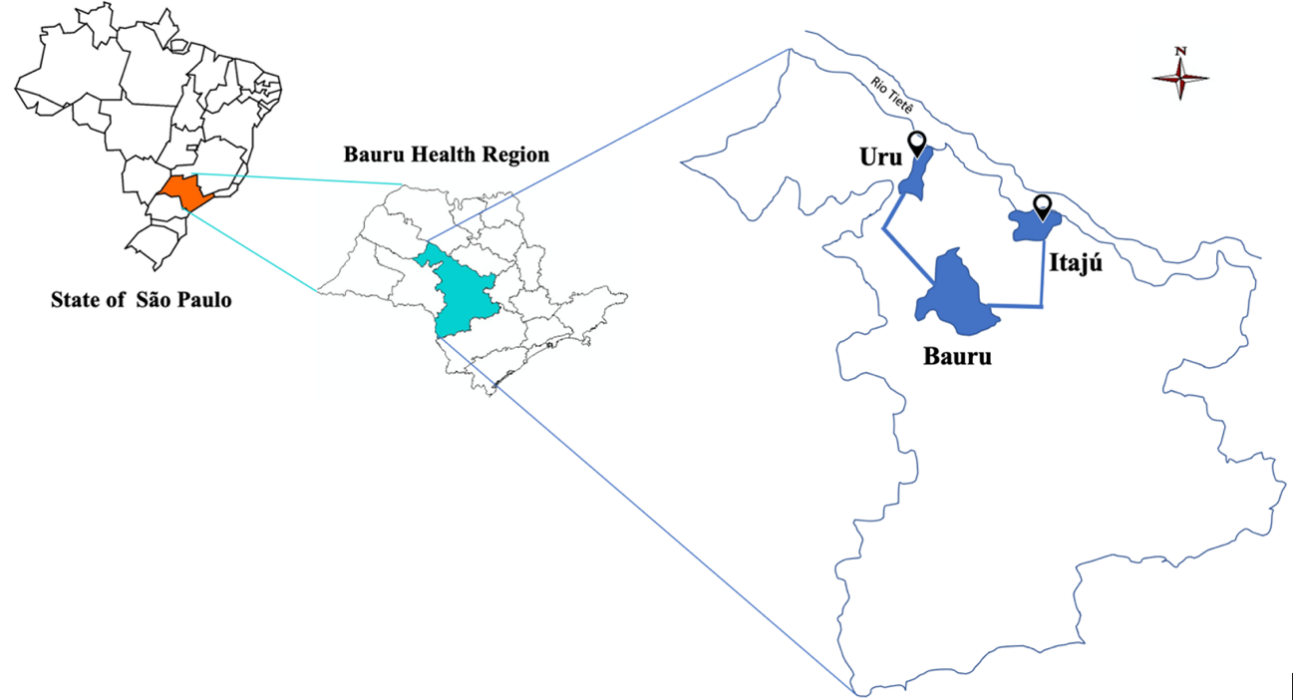
Map of the Bauru regional health, test and control community (adapted from Seade foundation; SEADE)

The test and control communities included fewer than 5,000 inhabitants, between 70 and 85% living in urban areas, with a demographic density of 7–15 inhab./km^2^, municipal human development index=0.7 and proportional labor force population of 15%–30%. In addition, these two municipalities have only one access road and one local public health center (Figure 1 (Fig. 1)).

With the spread of the SARS-CoV-2, some protective measures were adopted by the São Paulo State government that were strictly followed by the Bauru Health Region authorities, as well as by both communities. Thus, quarantine measures were divided into four steps: Step 1 (red), considered a contamination period, allowed only essential services, in which businesses and schools were closed, and established mobility restrictions and mandatory social distancing; step 2 (orange), considered an restricted opening period with the possibility of allowing some services, such as commerce with 20% of usual occupancy capacity was allowed and restriction of opening days with implementation of standard protocols, such as the use of masks, alcohol gels for hand antisepsis, temperature measurement, and social distancing; step 3 (yellow), considered a controlled period with some flexibility; and step 4 (green), considered a partial opening period, in which all services were allowed, respecting the 60% occupancy limit, maintaining all specific protocols [21], [22].

### Population mouthwash intervention protocol

The test community included all the residents in the urban households over 10 years old, who read and understood the risks and objectives of the study, signed the informed consent form, and received two bottles containing 600 mL of the APD mouthwash for use over a 2-month period. They were instructed to use 5 mL of the mouthwash and to switch between gargling/rinsing 3 to 5 times for 1 min per day. The orientation was provided in writing and explained by the researcher with her research assistant as many times as necessary for a period of 2 months, containing adequate information for the age groups of adults and children over 10 years old. During the 2-month period, the researcher was available to the population, who sought her out a few times to clarify doubts about the use of the product. Inhabitants who declared contraindications to using mouthwash for medical reasons or the inability to gargle and spit were excluded.

### Data sources and outcome measures

The study used official data from the Statewise System for Data Analysis maintained by the government of Sao Paulo state [23]. Information on cases and deaths included the place of residence of the patient. Consistency tests in the databases were performed daily, and their variations, characterized as outliers, were reported to the data collectors and reviewed in the calculations. Cases were considered confirmed for COVID-19 when a positive reverse-transcription polymerase chain reaction test result was obtained. The number of COVID-19 cases was divided into three phases according to the epidemiological week: 1: before the intervention (15^th^ to 47^th^ week); 2: during the intervention (48^th^ week of 2020 to 4^th^ week of 2021); 3: after intervention (5^th^ to 19^th^ week).

### Statistical analysis

Data were organized in a Microsoft Office Excel 2016 spreadsheet (Microsoft Corporation, Redmond, WA, USA), and analyses were conducted using the R Core Team program (2021). Incidences and risks of the disease in the three phases described for the control and test communities were calculated. Considering the size of populations under comparison, the relative risk (RR), standard error, and 95% confidence interval were estimated by subtracting the confirmed cases of the earlier phase in the denominator of the reference population. Differences at the 10% level were assumed to be significant because of the innovative features of the study. The null hypothesis (H0) proposed that there was no difference between the two municipalities regarding the RR.

## Results

**Figure 2:**
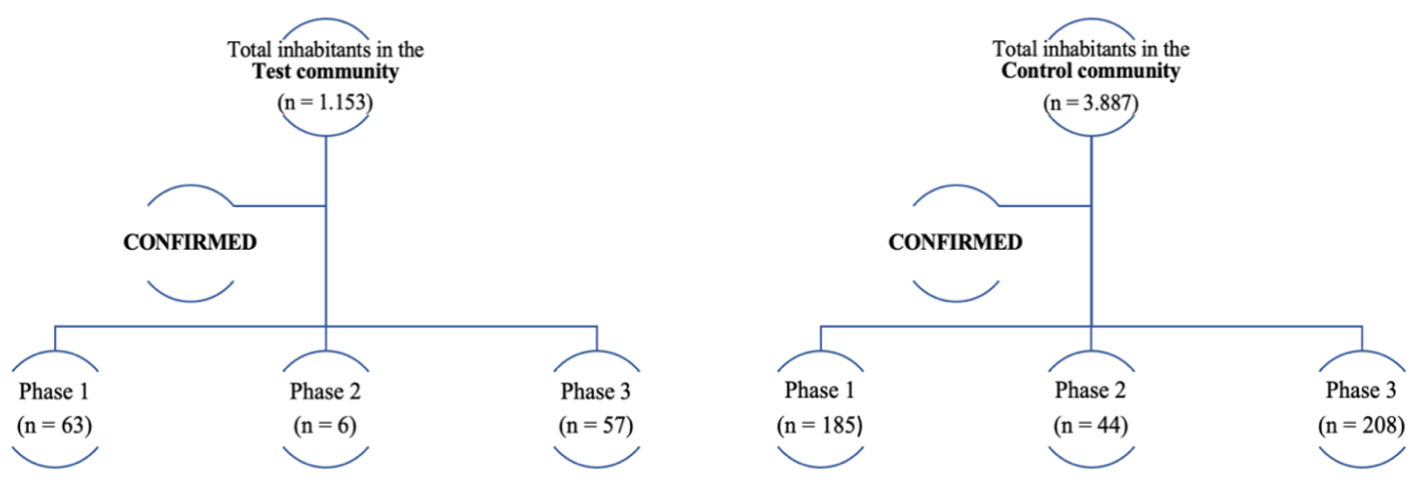
Flowchart of the reference population

**Figure 3:**
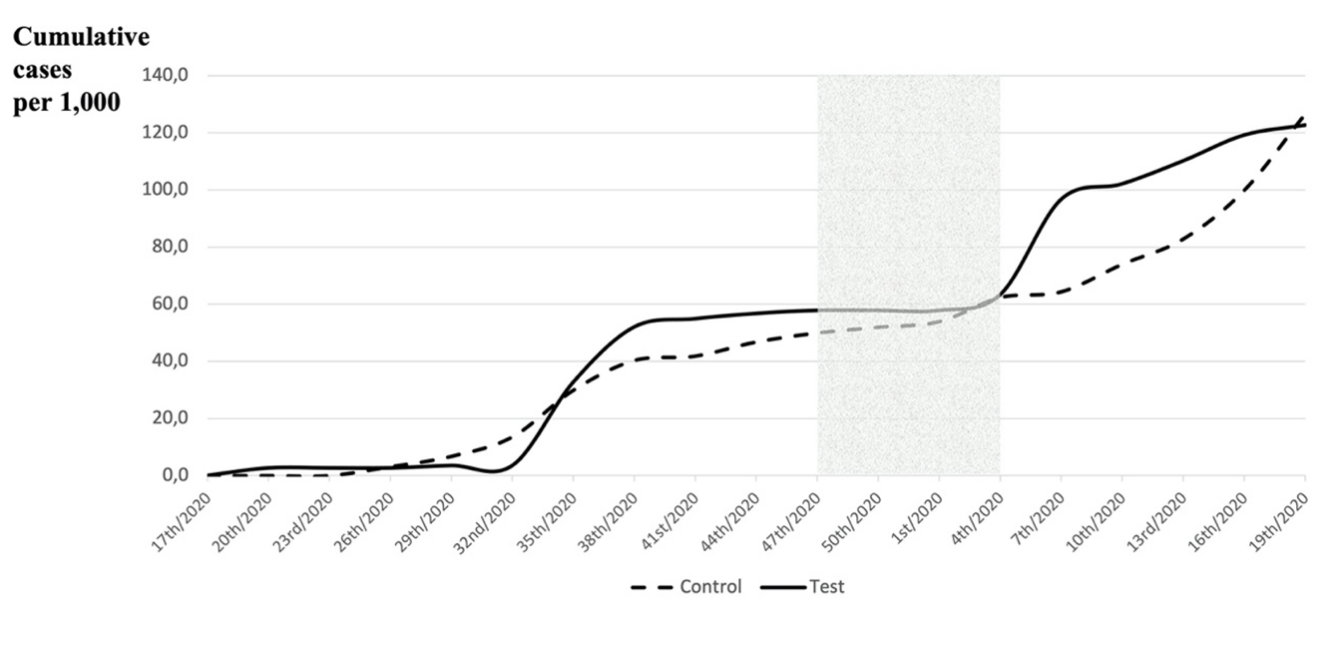
Cumulative incidence per 1,000 inhabitants in the test and control communities every three epidemiological weeks

Figure 2 (Fig. 2) illustrates the total number of confirmed cases of COVID-19 for the test and control communities before, during, and after the intervention. Figure 3 (Fig. 3) shows the curves of cumulative cases in each community every 3 epidemiological weeks. 

The gray area is related to the intervention period (phase 2). In phase 1, the increase in new cases began earlier in the control community (26^th^ week of 2020) than in the test community (32^nd^ week of 2020); however, since the 35^th^ week of 2020, cumulative cases in the test community surpassed those in the control community. In phase 2, the number of new cases increased more in the control community than in the test community. In phase 3, after the intervention period, the increase in new cumulative cases was similar between the 4^th^ and 16^th^ week of 2021. The curves of cumulative incidence of each community patterned every 3 weeks showed small differences in deflection related to the dynamics of epidemics in each community. It is noteworthy that the test community showed a different pattern in the intervention period (gray area of Figure 3 (Fig. 3)) and that both communities’ curves met each other at the end of the entire observation period.

In the period prior to the intervention (phase 1), the RR between the two municipalities was 1.15 (0.87–1.51) and did not significantly differ. The cumulative incidence of the test community was 54.6 per 1,000 residents, while it was 47.6 in the control community (p=0.331). In the intervention period (phase 2), the RR between the municipalities was 0.46 (0.20–1.08) with a statistically significant reduction in risk (-54.0%). The cumulative incidence in the test community was 5.2% per 1,000 residents, while in the control community it was 11.3% (p=0.076). In the period after the intervention (phase 3), the RR was 0.92 (0.69–1.23) and the cumulative incidences did not differ between the test and control communities (p=0.591) (Table 1 (Tab. 1)).

## Discussion

The main finding of this study was that the cumulative incidence of COVID-19 in the test community was lower than that in the control community during the use of a population-based virucidal phthalocyanine gargling/rinsing protocol. This is a promising result, as COVID-19 is very recent, and there is still no “gold standard” treatment; thus, actions based on primary prevention measures to slow down and interrupt transmission are essential. Populations pre-experienced in viral epidemics adopted now-proven preventive measures with risk reduction and benefits, such as social distancing, hand hygiene, wearing masks, and gargling/rinsing with antiseptic solutions [24], [25], [26], [27]. Since saliva has been an important source of virus transmission during the pandemic, protective measures such as the use of antiseptic gargles against SARS-CoV-2 were adopted and recommended by several countries and organizations [5], [10], [26], [27], [28]. 

Some studies have shown that the use of a mouthwash containing APD can reduce the viral load of SARS-CoV-2, acting as a therapeutic aid in reducing the severity and risk of COVID-19 transmission [8], [9], [28], [29], [30]. These data corroborate the findings of the present study. The test and control municipalities did not differ in terms of disease risk before the distribution of mouthwashes (phase 1) (p>0.05); however, disease risk was lower in the test than in the control community after the widespread use of the APD mouthwash/gargling protocol.

COVID-19 is a disease with pandemic characteristics that spreads according to population mobility [22]. The municipalities selected in this community trial have similar population sizes and social indicators. They belong to the same geographic mesioregion, have a similar primary healthcare structure, and are connected with neighboring municipalities by a single road [31], [32]. In both communities, the measures to control the COVID-19 pandemic in the whole study period were similar regarding the recommendations for social distancing and self-administered hygiene measures, inducing people to wear face masks, frequently wash their hands with soap, and, if necessary, use alcohol gel on the hands. Therefore, there were no reasons for any incidence differences in COVID-19 cases between the two communities, except for the virucidal phthalocyanine gargling/rinsing protocol used in the test community. Based on these points, the comparison presented here is assumed to be legitimate.

The cumulative incidence difference of COVID-19 at the population level in the test community was nearly significant, showing promising findings. Prior to the beginning of the use of the APD mouthwash, the curves of cumulative COVID-19 cases in the two communities were visually similar; however, at the 38^th^ week, the test community’s curve seemed todeviate, with a small number of new cases, while still showing a slight increase. However, despite the continued increase in COVID-19 cases in the control community, in the test community, new cumulative cases per 1,000 inhabitants were lower than those in the control community. In phase 2, as soon as the population was exposede to APD mouthwash, the relative risk decreased to 0.46, suggesting a preventive effect in the test community. The p-value was slightly too high (p=0.076) to be considered significant, but sufficient to be considerable in an epidemiologic study during a very difficult moment in history. After phase 2, the number of new COVID-19 cases increased in both communities. After the intervention period, the test community data showed a new cumulative case curve similar to the control community, highlighting the potential effect of the APD mouthwash at the population level.

Saliva is a biological fluid important in the spread of COVID-19. The presence of SARS-CoV-2 in saliva causes viral proliferation and consequent RNA secretion in any cells/tissues involved in the production of salivary components, such as salivary glands, respiratory tract cells, and periodontal tissue [33], [34]. Although our research did not analyze the salivary components and substantivity, we observed a difference in COVID-19 risk in the test community compared to the control community. This was supported by the finding that the virus has been consistently detected in saliva; thus, the oral cavity is a source of SARS-CoV-2 [30]. Thus, rinsing/gargling with an APD solution could chemically/mechanically reduce the action of the virus in the oral cavity and throat. In parallel, a virucidal nasal spray should be used, because the viral load in nasopharyngeal swabs is higher than in saliva [35], so that both areas must be included in virucidal antisepsis to protect the upper airways.

Strengths and limitations of this study must be considered in relation to the methodology used. The study was undertaken at a challenging historic moment, and the field team did not have the conditions to register the use of the APD mouthwash. Thus, important variables, such as personal/family behavior related to COVID-19 could not be obtained to control for possible confounding factors. In contrast, all the assessments were conducted at the population level, without any interference from the research group regarding each case and were publicly registered in both communities. The widespread use of masks, which could also have contributed to reducing transmission of COVID-19, was not assessed in this study. A difference in COVID-19 cumulative cases was observed in the current study, suggesting an effect of the intervention and possible contribution to keeping the health system from collapsing while saving lives.

## Conclusions

The use of the APD mouthwash protocol seems to reduce the COVID-19 incidence at the population level, but further studies are needed to confirm its protective effect in different contexts.

## Notes

### Funding source

This research was funded primarily by TRIALS – Oral Health & Technologies. Funders contributed to the scope and design of this study; however, they did not influence the collation, management, analysis, and interpretation of the data; preparation, review, or approval of the manuscript; or the decision to submit the manuscript for publication. This study was financed in part by the Coordenação de Aperfeiçoamento de Pessoal de Nível Superior (CAPES), Brazil (Finance Code 001). Dr. Da Silva Santos reports from CNPq process nº. 309525/2018-7. Dr. Frazão received a scholarship from CNPq process nº. 305132/2019-9.

### Acknowledgments

We acknowledge TRIALS – Oral Health & Technologies and the Coordenação de Aperfeiçoamento de Pessoal de Nível Superior (CAPES), Brazil, and thank Magali de Brito, the study’s research assistant.

### Competing interests

The authors declare that they have no competing interests.

## Figures and Tables

**Table 1:**
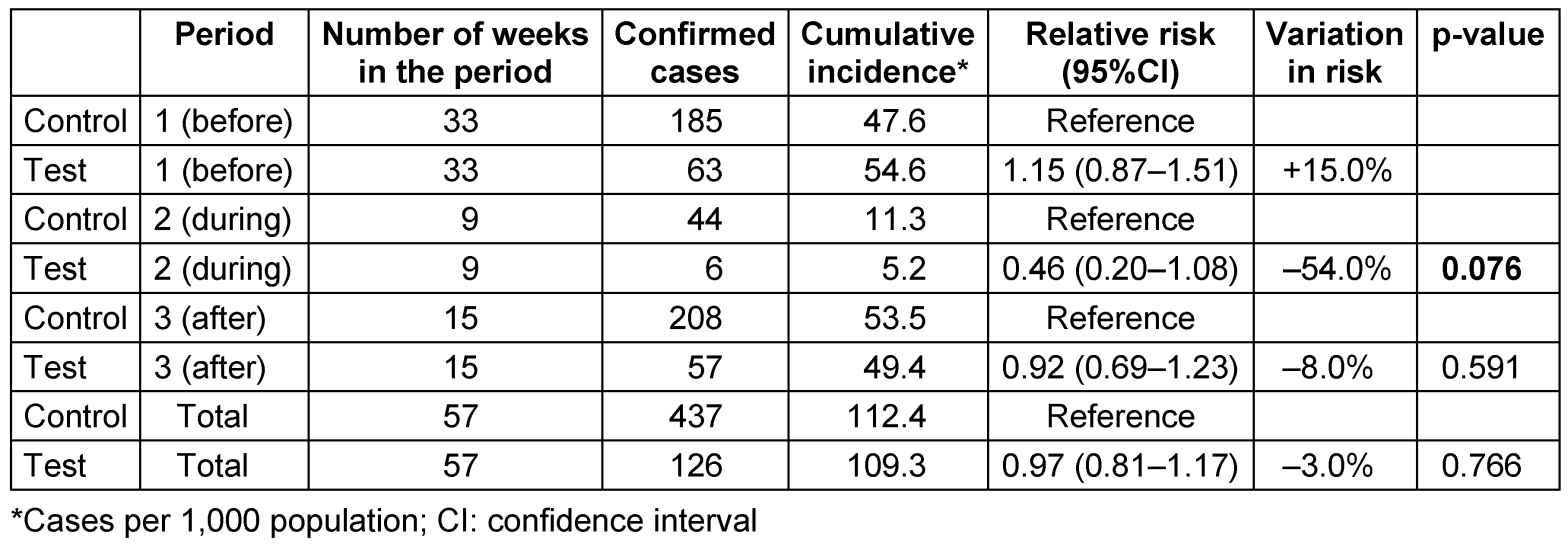
Cumulative incidence values and relative risk of coronavirus disease-2019 cases between the test and control communities in each evaluated period
